# Supplementary material for: Posterior Tibial Nerve Stimulation in Children with Lower Urinary Tract Dysfunction: A Mixed-Methods Analysis of Experiences, Quality of Life and Treatment Effect
Source: Int J Environ Res Public Health. 2022 Jul 25;19(15):9062. doi: 10.3390/ijerph19159062 (PMC9331059; doi:10.3390/ijerph19159062)
Supplement: Supplementary file 1 [file ijerph-19-09062-s001.zip › PTNS_COREQ_.pdf]

Supplemental data: COREQ checklist

Consolidated criteria for reporting qualitative studies (COREQ): 32-item checklist

Developed from:
Tong A, Sainsbury P, Craig J. Consolidated criteria for reporting qualitative research (COREQ): a 32-item checklist for interviews and focus groups. *International Journal for Quality in Health Care*. 2007. Volume 19, Number 6: pp. 349 – 357

No. Item	Guide questions/description	Our study
**Domain 1: Research team and reflexivity**		
Personal Characteristics		
1. Interviewer/ facilitator	Which author/s conducted the interview or focus group?	*Pluk Bekker*
2. Credentials	What were the researcher's credentials? E.g. PhD, MD	*MD (LW), BSc (CD), LO (MD), VJ (NP), BK (MD/PhD), JH (MD/PhD PhD (AO)*
3. Occupation	What was their occupation at the time of the study?	*Urologist (LW/ JH/ BK), Medical doctor (LO), student (PB), nurse practitioner (VJ) assistant professor (AO)*
4. Gender	Was the researcher male or female?	*All female except JH*
5. Experience and training	What experience or training did the researcher have?	*Short interview instruction, clinical counselling / communications training (PB/ LW), coding training and experience in coding (PB/ LW/AO) experienced interviewer (15+ years) (AO)*
Relationship with participants		
6. Relationship established	Was a relationship established prior to study commencement?	*Yes, LW/ BK were treating physicians. PB who performed the interviews had no relationship with the subjects*
7. Participant knowledge of the interviewer	What did the participants know about the researcher? e.g. personal goals, reasons for doing the research	*Reasons for doing the research*
8. Interviewer characteristics	What characteristics were reported about the inter viewer/facilitator? e.g. Bias, assumptions, reasons and interests in the research topic	*None*

Domain 2: study design		
Theoretical framework		
9. Methodological orientation and Theory	What methodological orientation was stated to underpin the study? e.g. grounded theory, discourse analysis, ethnography, phenomenology, content analysis	*This was an explorative study using a content analysis approach.*
Participant selection		
10. Sampling	How were participants selected? e.g. purposive, convenience, consecutive, snowball	*Purposive sampling*
11. Method of approach	How were participants approached? e.g. face-to-face, telephone, mail, email	*Contacted before or after treatment or email*
12. Sample size	How many participants were in the study?	*11*
13. Non-participation	How many people refused to participate or dropped out? Reasons?	*One parent. To busy at the time of the study*
Setting		
14. Setting of data collection	Where was the data collected? e.g. home, clinic, workplace	*At the hospital or online with video conferencing software*
15. Presence of non-participants	Was anyone else present besides the participants and researchers?	*No*
16. Description of sample	What are the important characteristics of the sample? e.g. demographic data, date	*All participants were parents of children with therapy-refractory urinary symptoms treated in a third line clinic. Variation in age, gender and different stages of their PTNS treatment (at start/ halfway or near the end or completed)*
Data collection		
17. Interview guide	Were questions, prompts, guides provided by the authors? Was it pilot tested?	*The authors designed an interview guide. The interview guide was modified with minor revisions of a few questions after the first interviews. No pilot study was performed*
18. Repeat interviews	Were repeat interviews carried out? If yes, how many?	*No*
19. Audio/visual recording	Did the research use audio or visual recording to collect the data?	*Audio*

20. Field notes	Were field notes made during and/or after the interview or focus group?	*Yes*
21. Duration	What was the duration of the inter views or focus group?	*Between 40 to 50 minutes*
22. Data saturation	Was data saturation discussed?	*Yes, data saturation was achieved*
23. Transcripts returned	Were transcripts returned to participants for comment and/or correction?	*No*
**Domain 3: analysis and findings**		
Data analysis		
24. Number of data coders	How many data coders coded the data?	*Two (PB and LW)*
25. Description of the coding tree	Did authors provide a description of the coding tree?	*No*
26. Derivation of themes	Were themes identified in advance or derived from the data?	*Derived from the data*
27. Software	What software, if applicable, was used to manage the data?	*ATLAS.ti*
28. Participant checking	Did participants provide feedback on the findings?	*No*
Reporting		
29. Quotations presented	Were participant quotations presented to illustrate the themes/findings? Was each quotation identified? e.g. participant number	*Participant quotations are present, and only identified by category (because of anonymity)*
30. Data and findings consistent	Was there consistency between the data presented and the findings?	*Yes*
31. Clarity of major themes	Were major themes clearly presented in the findings?	*Yes*
32. Clarity of minor themes	Is there a description of diverse cases or discussion of minor themes?	*Yes*
