# Supplementary material for: Posterior Tibial Nerve Stimulation in Children with Lower Urinary Tract Dysfunction: A Mixed-Methods Analysis of Experiences, Quality of Life and Treatment Effect
Source: Int J Environ Res Public Health. 2022 Jul 25;19(15):9062. doi: 10.3390/ijerph19159062 (PMC9331059; doi:10.3390/ijerph19159062)
Supplement: Supplementary file 1 [file ijerph-19-09062-s001.zip › Semi-structured interviews with parent (and child).pdf]

Semi-structured interviews with parent (and child)

Research question:

- How do parents and children experience the PTNS treatment?

Interview:

1. Introduction interviewer and research
2. Explanation interview
3. Questions:
    - How many PTNS session did [child] have?
    - Can you tell me more about the PTNS?
    - What were your expectations?
    - How did the first PTNS session go? How did it go in further sessions?
    - How does the PTNS feel for [child]?
    - Do you tell others about the PTNS and the urinary problem?
    - How do you experience the urinary problem?
    - What did you think of the other children being treated at the same time? What did you think of the contact with others?
    - What do you think of the contact with the healthcare provider providing PTNS?
    - How do you feel about answering questions in front of others?
    - How do you experience coming to the hospital every Friday for 12 weeks?
    - Were there negative experiences with the PTNS?
    - Are there effects of the PTNS treatment?
    - If you could change anything about PTNS treatment, what would it be?
4. Thanking and closing interview
